# Exploring new horizons in neovascular age-related macular degeneration: novel mechanisms of action and future therapeutic avenues

**DOI:** 10.1038/s41433-024-03373-x

**Published:** 2024-10-08

**Authors:** Jonathan D. Shirian, Priya Shukla, Rishi P. Singh

**Affiliations:** 1 Case Western Reserve University School of Medicine, Cleveland, OH USA; 2 Center for Ophthalmic Bioinformatics, Cole Eye Institute, Cleveland Clinic, Cleveland, OH USA; 3 Cleveland Clinic Lerner College of Medicine of Case Western Reserve University, Cleveland, OH USA; 4 Cole Eye Institute, Cleveland Clinic Foundation, Cleveland, OH USA; 5 Cleveland Clinic Martin Health, Cleveland Clinic Florida, Stuart, FL USA

**Keywords:** Drug therapy, Antibody therapy

## Abstract

Neovascular age-related macular degeneration (nAMD) can lead to significant vision impairment through the growth of abnormal neovascular membranes in the choroid. Despite advancements with current anti-vascular endothelial growth factor (VEGF) therapies, challenges such as frequent injections, inadequate response, and patient-related concerns persist. Emerging therapeutics aim to reduce vision-loss through a variety of mechanisms. Gene therapies, including RGX-314 and Ixo-vec, express an anti-VEGF protein, and 4D-150, expresses an anti-VEGF protein and a VEGF-C inhibitory miRNA. Anti-VEGF associated therapeutics include OPT-302, targeting VEGF-C and VEGF-D, BI 836880, which inhibits VEGF-A and Ang-2 activity, and Tarcocimab tedromer, inhibiting all VEGF-A isoforms. Agents with novel mechanisms of action include UBX1325, which inhibits an anti-apoptotic protein, Restoret (EYE103), a Wnt agonist, and the tyrosine kinase inhibitors, EYP-1901, OTX-TKI, CLS-AX, and KHK4951.

## Abstract (zh)

新生血管性年龄相关性黄斑变性(nAMD)因脉络膜异常新生血管膜的生长造成严重的视力损害。尽管目前抗血管内皮生长因子 (VEGF) 在治疗方面取得很大进展, 但频繁注射、应答不佳及患者相关问题等挑战依然存在。新兴疗法旨在通过多种作用机制来减少视力丧失。基因治疗包括RGX-314和Ixo-vec表达抗 VEGF 蛋白, 4D-150 表达抗 VEGF 蛋白和抑制 miRNA 的VEGF-C。抗VEGF相关疗法包括针对VEGF-C和VEGF-D的OPT-302, 以及抑制 VEGF-A和Ang-2活性的BI 836880。新的作用机制药物有抑制抗凋亡蛋白Restoret(EYE103)、Wnt激动剂UBX1325以及酪氨酸激酶抑制剂EYP-1901和OTX-TKI。

## Introduction

Age-related macular degeneration (AMD) poses a significant global health challenge, standing as a leading cause of blindness across many developed nations [1]. Approximately 288 million people worldwide are projected to have AMD by 2040, as the world’s population is growing older and it is a disease that disproportionately affects elder individuals [2, 3]. AMD encompasses two main subtypes: non-neovascular AMD and nAMD. While the non-neovascular form accounts for the majority of cases, nAMD is responsible for approximately 80% of severe vision loss associated with AMD [4]. nAMD is characterized by the growth of pathologic choroidal neovascular membranes (CNVM) which lead to haemorrhage and exudation in the retina. This abnormal vascular growth is driven by the upregulation of VEGF, a growth factor involved in angiogenesis and vascular permeability [5].

The introduction of intravitreally administered anti-VEGF agents marked a significant breakthrough in the treatment of nAMD. Previous treatments, including photodynamic therapy with verteporfin, intravitreal steroids, focal laser therapy, and surgical excision of CNVMs, did not improve vision and grew obsolete [6]. Pegaptanib (Macugen, Pfizer) was the first FDA-approved anti-VEGF agent in 2004, however, with the introduction of other increasingly effective anti-VEGF therapies, the utilization of this drug has significantly diminished. Today, the most commonly used agents to treat nAMD include bevacizumab, ranibizumab, aflibercept, brolucizumab, and faricimab (Table 1). Brolucizumab (Beovu, Novartis) was FDA-approved to treat nAMD in 2019 but recent concerns regarding related retinal vasculitis and intraocular inflammation have raised caution around its use [7]. Faricimab (Vabysmo, Roche/Genentech) was most recently approved in 2022 and is the only agent which targets both VEGF-A and angiopoietin-2 (Ang-2).

**Table 1:** Current approved nAMD therapies.

Name	Molecular target	Structure	Year approved	Company	Dosing frequency	Molecular weight
Pegaptanib (Macugen)	Anti-VEGF-A	Small oligonucleic acid molecule	FDA: 2004 EMA: 2006 MHRA: 2006	OSI Pharmaceuticals/ Pfizer	6 weeks	50 kDa
Bevacizumab (Avastin)	Anti-VEGF-A	IgG1	FDA: 2004 EMA: 2005 MHRA: 2005	Genentech/Roche	4–8 weeks	149 kDa
Ranibizumab (Lucentis)	Anti-VEGF-A	Fab fragment	FDA: 2006 EMA: 2007 MHRA: 2007	Genentech/ Novartis	4–8 weeks	48 kDa
Aflibercept 2 mg (Eylea)	Anti-VEGF-A Anti-VEGF-B Anti-PGF	IgG Fc + VEGFR1/2 fusion protein	FDA: 2011 EMA: 2012 MHRA: 2012	Regeneron/Bayer	8 weeks	115 kDa
Aflibercept 8 mg (Eylea HD)	Anti-VEGF-A Anti-VEGF-B Anti-PGF	IgG Fc + VEGFR1/2 fusion protein	FDA: 2023 EMA: 2023 MHRA: 2024	Regeneron/Bayer	8–16 weeks	115 kDa
Brolucizumab (Beovu)	Anti-VEGF-A	Single-chain variable fragment	FDA: 2019 EMA: 2020 MHRA: 2020	Novartis	8–12 weeks	26 kDa
Faricimab (Vabysmo)	Anti-VEGF-A Anti-Ang-2	Bi-specific antibody	FDA: 2022 EMA: 2022 MHRA: 2022	Genentech/Roche	12–16 weeks	149 kDa

However, despite these advancements, the current standard of care presents practical limitations, including high treatment costs, the need for frequent intravitreal injections, intravitreal-injection related fear and anxiety, and transportation issues [8, 9]. These factors hinder adherence to treatment regimens leading to a 2-year loss to follow-up rate of 28% and a 5-year loss to follow-up rate of 57% [10, 11]. Additionally, there are concerns related to treatment response, such as non-responders and the development of adverse outcomes like fibrosis, which impact visual improvement [12]. For example, in the MARINA and ANCHOR clinical trials, approximately 10% of patients treated with ranibizumab experienced a decrease in visual acuity of 15 letters or more from baseline, highlighting the variability in patient response to anti-VEGF therapy [13]. The limitations of current nAMD therapies have prompted a growing interest in the development of novel agents and treatment modalities. Researchers are exploring alternative pathways and targeting molecules beyond VEGF to improve outcomes and reduce the burden of frequent injections. This review article will explore the current therapeutic landscape of nAMD by describing ongoing clinical trials and emerging therapies, including gene therapy, combination anti-VEGF agents, and novel mechanisms of action.

## Methods

Therapeutics included in this review were identified through a search of ClinicalTrials.gov with the search terms “Neovascular Age-Related Macular Degeneration” and “Wet Age-related Macular Degeneration.” Inclusion criteria include: therapeutics with novel mechanisms of action to nAMD treatment in phase 1–3 clinical trials. Exclusion criteria include: no clinical trial results, sustained delivery systems of existing therapeutics, therapeutics exclusively targeting complement, and anti-VEGF biosimilars. Data was obtained in January 2024.

### Gene therapy

RGX-314 (REGENXBIO, Rockville, Maryland) is a promising gene therapy for nAMD that utilizes an adeno-associated virus serotype 8 (AAV8) vector expressing a ranibizumab like anti-VEGF Fab (Table 2). A phase I/IIa open-label dose-escalation study (*n* = 42), compared five cohorts receiving escalating doses of subretinal RGX-314. RGX-314 was found to be generally well–tolerated across all dose levels with one possible drug-related serious adverse event (SAE) of significantly decreased visual acuity in a patient in the highest dose cohort [14, 15]. Patients receiving the second highest and highest dose level in this study experienced a 58.3% and 81.2% reduction in anti-VEGF injection rate over 1.5 years, respectively [14]. Interim data from the phase II AAVIATE® trial (NCT04514653) utilizing a suprachoroidal delivery method demonstrates that RGX-314 continues to be well tolerated among the 106 patients enroled in its three dose levels with no drug-related SAEs [16]. At the highest dose level, 50% of patients remain injection-free after RGX-314 administration and experience an overall 80% reduction in injection rate each year, while maintaining a stable best-corrected visual acuity (BCVA). Ongoing trials, ATMOSPHERE® (phase IIb/III, NCT04704921) and ASCENT® (phase III, NCT05407636), are further evaluating subretinal delivery of RGX-314.

**Table 2:** Future therapeutic agents.

Class	Name	Structure	Target	Method of delivery
Gene therapy	RGX-314	AAV8 vector expressing a ranibizumab like Fab	VEGF-A	Subretinal/Suprachoroidal
	Ixoberogene soroparvovec (Ixo-vec)	AAV.7m8 vector encoding the aflibercept protein	VEGF-A, VEGF-B, PGF	IVT Injection
	4D-150	AAV vector (R100) expressing both aflibercept and a VEGF-C inhibitory miRNA	VEGF-A, VEGF-B, VEGF-C, PGF	IVT Injection
Anti-VEGF related	OPT-302	Recombinant fusion “trap” protein consisting of three extracellular ligand-binding domains of VEGFR-3	VEGF-C and VEGF-D	IVT Injection
	BI 836880	Bispecific nanobody®	VEGF-A, Ang-2	IVT Injection
	Tarcocimab tedromer (KSI-301)	IgG1 anti-VEGF antibody biopolymer conjugate	VEGF-A	IVT Injection
Tyrosine kinase inhibitor	EYP-1901	Selective tyrosine kinase inhibitor	VEGF-A, VEGF-B, VEGF-C, PDGF	IVT Injection
	OTX-TKI	Small molecule tyrosine kinase inhibitor	VEGFR-1, 2, and 3	Intravitreal bioresorbable hydrogel implant
	CLS-AX	Small molecule tyrosine kinase inhibitor	VEGFR-1, 2, and 3	Suprachoroidal space microinjector
	KHK4951	Small molecule tyrosine kinase inhibitor	VEGFR-1, 2, and 3	Eye drop
Other	UBX1325	Small molecule inhibitor	Bcl-xL	IVT Injection
	Restoret (EYE103)	Tri-specific Wnt agonist antibody	VEGFR-1, 2, and 3	IVT Injection

Ixoberogene soroparvovec (Ixo-vec) (Adverum Biotechnologies, Redwood City, California), previously known as ADVM-022, features an intravitreally (IVT) injected proprietary vector capsid, AAV.7m8, carrying a ubiquitous expression cassette encoding the aflibercept protein [17]. In the OPTIC phase I trial (NCT03748784), Ixo-vec demonstrated an acceptable safety profile with the most common drug-related adverse event being ocular inflammation (60%; *n* = 18) which responded well to topical corticosteroids [18, 19]. Key data from OPTIC revealed sustained aqueous humour levels of aflibercept over 96 weeks, leading to a reduction in the annualized anti-VEGF injection rate of 81% in the low-dose group and 98% in the high-dose group [18]. By 2 years, 53% of patients in the low-dose group and 80% in the high-dose group remained free from additional anti-VEGF injections. The phase II LUNA trial (NCT05536973) assessing Ixo-vec’s safety and efficacy is currently active, but not yet recruiting with an enrolment goal of 72 patients.

4D-150 (4D Molecular Therapeutics (4DMT), Emeryville, California) utilizes a proprietary AAV vector, R100, which can infiltrate the internal limiting membrane to increase retinal transgene expression. The vector carries a transgene cassette that expresses both aflibercept and a VEGF-C inhibitory miRNA, effectively inhibiting VEGF A, B, C, and placental growth factor. Interim results from its phase I/II trial, PRISM (NCT05197270), demonstrate 4D-150’s safety with no drug-related SAEs or dose-limiting toxicities [20]. At 36 weeks, 80% of participants did not require additional supplemental anti-VEGF injections with a 97.6% decline in the annualized anti-VEGF injection rate [20]. The FDA recently granted 4D-150 Regenerative Medicine Advanced Therapy designation, a first for any nAMD targeted therapy [21]. Collaborating with both the FDA and EMA, 4DMT is now working on preliminary phase III clinical trial plans.

### Anti-VEGF related

OPT-302 (Opthea limited, Victoria Australia) is a novel recombinant fusion “trap” protein consisting of three extracellular ligand-binding domains of human VEGF receptor(VEGFR)-3 fused to the constant domain of human immunoglobulin G1 (Table 2) [22, 23]. It specifically binds to and sequesters VEGF-C and VEGF-D, preventing their binding to VEGFR-2 and VEGFR-3 [22]. Intended to be administered via IVT injection along with traditional anti-VEGF agents that selectively target VEGF-A, a phase IIb dose-ranging study (NCT03345082) found that 2.0 mg OPT-302 in addition to ranibizumab generated substantial increases in visual acuity compared to ranibizumab alone [22]. OPT-302 produced anatomic improvements (a decrease in central subfield thickness (CST) of 14  μm and 12.9 μm in 0.5 mg and 2 mg groups, respectively) when compared with sham and was also found to have a favourable safety profile, with no significant differences across groups [22]. OPT-302 is currently undergoing phase III trials, building upon the promising phase IIb results. The ShORe (NCT04757610) and COAST (NCT04757636) trials aim to further assess the efficacy and safety of OPT-302 along with ranibizumab and aflibercept, respectively.

BI 836880 (Boehringer Ingelheim, Germany) is an IVT delivered humanized bispecific nanobody® designed to strongly bind to and inhibit the activity of VEGF-A and Ang-2 [24]. An additional albumin molecule is present on the nanobody, which extends its half-life post-injection [25]. A phase I/IIa trial (NCT03861234) revealed no drug-related or dose-limiting adverse events and demonstrated preliminary signs of efficacy in the improvement of BCVA [26]. Further investigations to explore the potential of BI 836880 are not yet active.

Tarcocimab tedromer (KSI-301) (Kodiak Sciences Inc., Palo Alto, California) is an IgG1 anti-VEGF antibody biopolymer conjugate targeting all VEGF-A isoforms, administered via IVT injection [27]. The DAYLIGHT phase III clinical trial (NCT04964089) demonstrated that monthly injections of Tarcocimab tedromer resulted in non-inferior improvements in BCVA at one year compared to aflibercept (3.9 vs. 5.5 ETDRS letters), with a similar decrease in CST (117 μm vs. 109 μm) [27]. Its safety profile was favourable, exhibiting low rates of intraocular inflammation (3.3%, *n* = 276) and no cases of intraocular inflammation with vasculitis or vascular occlusion. Tarcocimab tedromer will be further investigated in the DAYBREAK trial, which has an enrolment target of mid-2024.

### Tyrosine kinase inhibitor

EYP-1901 (EyePoint Pharmaceuticals, Watertown, Massachusetts) combines EyePoint’s proprietary bioerodible Durasert^®^ delivery technology with vorolanib, a selective tyrosine kinase inhibitor, which is a potent pan-VEGFR and platelet-derived growth factor receptor inhibitor (Table 2) [28]. In the phase I DAVIO trial (NCT04747197), EYP-1901 demonstrated positive twelve-month safety data with no reports of drug-related ocular or systemic SAEs and no dose-limiting toxicities [29]. The trial showed that after one dose 53% of patients did not need rescue anti-VEGF injections at 6 months [29]. Interim data from the phase II DAVIO 2 trial (NCT05381948) indicate that it met its primary endpoint, with both of the EYP-1901 doses utilized demonstrating non-inferiority in the change in BCVA compared to the aflibercept control [30]. The trial also achieved key secondary endpoints, including an over 80% reduction in anti-VEGF injection rate and almost two-thirds of patients not requiring additional anti-VEGF injections for up to six months [30].

OTX-TKI (Ocular Therapeutix, Bedford, Massachusetts) is an intravitreal bioresorbable hydrogel implant incorporating axitinib, a small molecule tyrosine kinase inhibitor. Axitinib is a selective and potent inhibitor of VEGFR-1, VEGFR-2, and VEGFR-3 and is currently FDA approved for the treatment of advanced renal cell carcinoma [31]. The hydrogel implant maintains therapeutic levels of axitinib, which is slowly diffused through the hydrogel network. The current phase I trial (NCT04989699) has produced positive 12-month results. OTX-TKI sustained visual acuity at levels similar to those achieved with aflibercept administered every eight weeks, resulting in an 89% reduction in anti-VEGF injection rate [32]. The therapeutic showed no drug-related ocular nor systemic SAEs [32]. Additionally, the observed consistent bioresorption of the implant at around nine months in the trial provides valuable insights into a potential redosing timeline. A phase III trial with an enrolment goal of 300 patients is set to begin shortly.

CLS-AX (Clearside Biomedical, Inc.) utilizes a proprietary suprachoroidal space microinjector to deliver a suspension of axitinib. The phase I OASIS trial (NCT04626128) and its 12-week extension study demonstrated an excellent safety profile in all dose cohorts with no SAEs and no adverse events from inflammation [33]. Through the extension study, the trial resulted in a 77% decrease in treatment burden with 50% of patients not requiring additional therapy at 6 months and stable BCVAs and CSTs [33]. A phase IIb clinical trial, ODYSSEY (NCT05891548), is currently active comparing CLS-AX with Faricimab, with topline data anticipated in the third quarter of 2024.

KHK4951 (Tivozanib) (Kyowa Kirin Co., Ltd.) is a small-molecule tyrosine kinase inhibitor targeting VEGFR-1, -2, and -3 [34]. Tivozanib is currently FDA approved for the treatment of relapsed or refractory advanced renal cell carcinoma. In this formulation it is administered as an eye drop and features nano-crystallized tivozanib, designed for efficient delivery to posterior ocular tissues. In a phase I clinical trial (NCT04594681), the therapeutic successfully met its safety outcome with a minimal number of adverse events. Currently, a phase II trial (NCT06116890) is actively recruiting patients to further evaluate its efficacy and safety.

### Additional targets

UBX1325 (Unity Biotechnology, South San Francisco, California) is a small molecule inhibitor of Bcl-xL, an anti-apoptotic protein within the Bcl-2 family (Table 2) [35]. It promotes apoptosis of senescent vascular endothelial cells which secrete VEGF, thereby exhibiting anti-VEGF properties [36]. While its phase II ENVISION study (NCT05275205) exhibited a favourable safety and tolerability profile, UBX1325 did not meet the non-inferiority margin at 24 weeks compared to aflibercept [35, 37]. Despite this, 52% of treated participants did not require a supplemental anti-VEGF injection at 24 weeks [37]. Further exploration of UBX1325 is being conducted in diabetic macular oedema, with a phase IIb trial (NCT06011798) actively recruiting.

Restoret (EYE103) (Eyebiotech Limited, United Kingdom) is a tri-specific Wnt agonist antibody delivered by IVT injection. Restoret mimics the natural ligand Norrin, which agonizes the Wnt signalling pathway [5, 38]. Norrin is crucial in restoring and maintaining the blood-retinal barrier by inducing tight junction organization, limiting vascular permeability [5, 39]. The phase Ib/IIa AMARONE (Anti-permeability Mechanism and Age-Related Ocular Neovascularization Evaluation) clinical trial (NCT05919693) is a two-part study, comprising an open-label multiple ascending dose safety study and a dose-finding single-masked comparative safety and preliminary efficacy study of IVT Restoret. Preliminary 12-week data from this study revealed no drug-related SAEs and that Restoret was well tolerated [38]. Eyebiotech Limited also reported an increase in BCVA of +11.2 letters and a decrease in retinal thickness of 143 microns among patients in the diabetic macular oedema arm of the study (*n* = 26) with similar results in those in the nAMD arm (*n* = 5) who received combination therapy with aflibercept [38].

## Conclusion

In conclusion, nAMD continues to present a substantial global health challenge with a growing prevalence projected for the future. The advent of IVT administered anti-VEGF agents marked a breakthrough, significantly improving outcomes compared to previous modalities. However, the current standard of care for nAMD poses the previously mentioned practical limitations and efficacy issues.

The pursuit of therapies that address the multifaceted challenges of nAMD remains crucial. The development of longer-lasting drugs, gene therapy, and alternative pathways reflects a shift towards enhancing patient satisfaction and compliance and reducing the overall management burden associated with nAMD. While the investigational landscape for nAMD is promising (Table 2), it is essential to acknowledge the high standard set by current anti-VEGF therapies. Future advancements should aim not only for equivalent or superior efficacy but also for improved safety, tolerability, and convenience. The complexity of nAMD pathogenesis demands a multifaceted approach, and ongoing research holds the potential to redefine the treatment paradigm for this visually debilitating condition.
